# A stable tensorial approach for inversion of adsorption potential energy from isotherm data

**DOI:** 10.1007/s00894-026-06963-6

**Published:** 2026-09-28

**Authors:** Márcio Oliveira Alves, Felipe Silva Carvalho, João Pedro Braga

**Affiliations:** 1 Departamento de Química, Centro Federal de Tecnologia de Minas Gerais, Av. Amazonas 5253, Belo Horizonte, 30421-169 Minas Gerais Brazil; 2 Division of Chemistry and Chemical Engineering, California Institute of Technology, 1200 East California Boulevard, Pasadena, 91125 CA USA; 3 Departmento de Química, Universidade Federal de Minas Gerais, Av. Presidente Antônio Carlos 6627, Belo Horizonte, 31270-901 Minas Gerais Brazil

**Keywords:** Adsorption, Potential energy, Tensorial contraction, Inverse problem

## Abstract

****Context**:**

Underlying the adsorption mechanism is not a simple task due to the complexity of the process. In this context, statistical mechanics provides a versatile approach with relatively low computational cost for describing adsorption phenomena. An alternative strategy consists of obtaining the adsorption potential energy from experimental data through the solution of an inverse problem. In this work, the adsorption potential energy was determined directly from adsorption isotherms using experimental datasets for both aqueous- and gas-phase adsorption systems. The proposed methodology was compared with the traditional two-step procedure, in which the second adsorption virial coefficient is first determined and subsequently used for inversion of the potential energy. Due to the ill-posed nature of the problem, the conventional approach exhibits significant instability and error propagation. In contrast, the direct inversion method provides excellent results, achieving values of $R^2 \sim 0.99$.

****Methods**:**

A methodology grounded in statistical physics was developed to obtain the adsorption potential energy directly from isotherm data. The proposed approach formulates the problem as a tensor contraction, eliminating the need for the traditional two-step procedure involving the intermediate determination of the second adsorption virial coefficient.

## Introduction

Adsorption is an efficient, versatile, and cost-effective method for removing contaminants from water and wastewater. Therefore, developing high-performance adsorbent materials is essential to improve removal efficiency. These materials have increased adsorption capacity, enabling faster and more effective treatment. In addition, advances in adsorbent design contribute to more sustainable and economical solutions, benefiting both the environment and water resource management [1–3].

Despite the widespread use of adsorption, achieving a comprehensive understanding of its mechanisms remains a challenge due to the complexity of the process. This complexity arises from the numerous sub-processes and intricate interactions between the adsorbent, adsorbate, and solvent [4]. Adsorption studies are typically conducted experimentally to evaluate equilibrium isotherms and process kinetics. These experiments provide valuable data for understanding how variables such as temperature, concentration, and time affect adsorption behavior [5, 6].

In a theoretical approach, advanced techniques such as electronic structure calculations [7], molecular dynamics simulations [8], and transport property studies [9] can be employed. In particular, electronic structure calculations, often based on methods such as density functional theory (DFT), allow the precise determination of energy levels and interactions within the adsorbate-adsorbent, providing valuable information on adsorption energies and geometric configurations. While experimental isotherms are often analyzed using empirical models such as Langmuir and Freundlich, these do not provide the detailed physical insight required to fully understand the adsorption process [10]. On the other hand, quantum chemical methods and molecular dynamics simulations are computationally demanding and may not provide an accurate description of adsorption on heterogeneous and porous surfaces [11].

An alternative approach consists in employing models based on statistical mechanics, which provide valuable insights into the adsorption process while maintaining a low computational cost. Ben Lamine et al. proposed a more realistic framework for studying adsorption phenomena based on Hill’s adsorption model [12, 13]. This method allows the application of a series of models to fit experimental adsorption isotherms and extract microscopic information, such as the number of layers, orientation anchorage, and number of adsorption sites. This approach has been demonstrated to be both powerful and versatile in describing adsorption processes across various adsorbates and adsorbents [14–18].

Another methodology grounded in statistical mechanics was introduced by Radke and Prausnitz [19]. Based on the theory of dilute solutions proposed by McMillan and Mayer [20], the adsorbent surface is treated as an external field influencing the solution. In this framework, the amount of material adsorbed on the surface is described by a virial expansion, in which each virial coefficient depends on the interaction potential between the adsorbed molecules and the surface. This formulation is consistent with Henry’s law, since the second virial coefficient (SVC) can be obtained from the slope of the isotherm at low concentrations. Consequently, if the adsorption potential energy (APE) is known, the SVC can be determined, allowing direct calculation of the adsorption isotherm. Conversely, determining the APE from experimental SVC data constitutes an inverse problem [21].

Experimental data inherently contain noise due to measurement errors. As a result, the inverse problem is ill-posed, meaning that the solution may be non-unique, unstable with respect to the data, or may not exist [22]. To address such issues, methods such as singular value decomposition [23], Tikhonov regularization [24], and Hopfield neural networks [25] are commonly employed. In a recent study, we applied Hopfield neural network to derive the APE from SVC data [26].

Since experimental adsorption isotherms are widely used in adsorption studies, an important approach is to determine the APE directly from these data. In this work, a generalized methodology is introduced to obtain the APE directly from adsorption isotherms, eliminating the need to first determine the SVC. To this end, a novel framework is proposed in which the inverse problem is formulated as a tensor contraction in a multiway setting, enabling direct estimation of the APE from isotherm data. The methodology is validated using different experimental datasets for both liquid- and gas-phase adsorption. By improving the generality and robustness of APE determination, this approach provides a powerful tool for predicting adsorption energies from experimental data.

## Methodology

### Thermodynamic background

The isotherm data for a very dilute solution near a solid surface can be represented as a virial series expansion [19]

1 $$\begin{aligned} Q_a=B_1(\mu ,T)c+C_2(\mu ,T)c^2+D_3(\mu ,T)c^3+... \end{aligned}$$

It is assumed that $Q_a$ represents the amount adsorbed on the surface and *c* is the concentration of the solution. The coefficients $B_1$, $C_2$, and $C_3$ are the virial coefficients, which are functions of the temperature *T* and the chemical potential μ. The coefficient $B_1$, corresponding to the second virial coefficient (SVC), is related to the interaction potential energy between a single adsorbed particle and the solid surface. The higher-order terms account for interactions involving two or more particles with the surface. The $B_1$ term is also associated with Henry’s constant of adsorption, since the isotherm becomes linear at low concentrations, where the higher-order terms in *c* vanish.

2 $$\begin{aligned} B_1=\lim _{c \rightarrow 0}\left( \frac{\partial Q_a}{\partial c} \right) _{T,\mu } \end{aligned}$$

The coefficient $B_1$ and the potential energy function are related by the following integral

3 $$\begin{aligned} B_1=\int _V[e^{-E_p(z)/k_BT}-1]dV \end{aligned}$$

where $k_B$ is the Boltzmann constant and $E_p(z)$ is the adsorption potential energy (APE) as a function of the perpendicular distance, *z*, between the particle and the surface. Alternatively, 3 can be rewritten by integrating over the *x* and *y* coordinates [26]:

4 $$\begin{aligned} B_1=A\int [e^{-E_p(z)/kT}-1]dz \end{aligned}$$

with the effective area of adsorption of a single particle in a very dilute solution denoted by *A*. The potential energy $E_p(z)$ can assume several functional forms, with the most commonly employed being the so called Lennard–Jones 10-4 potential.

5 $$\begin{aligned} E_p(z)=\left[ \frac{5(20)^{2/3}}{12}\right] e_0\left[ \left( \frac{d_0}{z}\right) ^{10}-\left( \frac{d_0}{z}\right) ^{4}\right] \end{aligned}$$

being $e_0$ the potential well depth and $d_0$ the distance for which the potential is zero. Equation 5 results from integrating the 12–6 potential between a particle and a planar surface.

### Direct and inverse problem formulation

The direct problem is easily solved as follows: 1) knowing the APE ($E_p(z)$) and an estimate for *A*, the SVC ($B_1$) is calculated by solving the integral in Eq. 4. Thus, the isotherm data can be determined using Eq. 1.

On the other hand, solving the inverse problem is a challenging task due to the ill-posed nature in the inversion of the potential energy in Eq. 3. Moreover, calculating $B_1$ from experimental isotherm data may lead to inaccurate determinations of the thermodynamic property. As indicated in Eq. 2, the value of $B_1$ can be derived from isotherms that exhibit linear behavior at low concentrations. However, extrapolating the derivative as c→0 often yields erroneous values due to inherent inaccuracies in the experimental data. In Radke’s work, the virial coefficient was determined through an empirical equation [27].

6 $$\begin{aligned} \frac{1}{Q}=\frac{1}{ac}+\frac{1}{b c^{\beta }} \end{aligned}$$

with $a=B_1$, *b* and β being empirical parameters obtained by a fitting procedure. Nevertheless, it is more appropriate to employ the method outlined in Eq. 1 to ascertain the second virial coefficient, since this method determines the coefficient through the fitting of a polynomial equation instead of a non-linear equation. Furthermore, Eq. 1 is based on the fundamental principles of thermodynamics, whereas Eq. 6 is an empirical equation. Once the correct value of $B_1$ has been determined, the potential energy $E_p(z)$ can be obtained through an inverse procedure that solves the nonlinear integral equation presented in Eq. 3. In other words, the inverse problem can be solved in two-step procedure.

To validate the robustness of this inversion methodology, we employ a self-consistency check: the obtained APE values are used to recalculate $B_1$, which is then applied along with the remaining model parameters to reconstruct the original isotherm. In this work, two distinct two-step procedures are employed to determine the APE from isotherm data. In Model 1, Eq. 6 is used, while Model 2 is based on Eq. 1 to determine $B_1$. The APE is then obtained in a subsequent step by inverting the SVC.

### Inversion procedure via tensorial representation

It is important to note that the process on Model 1 and Model 2 is susceptible to significant errors if the inversion in any of the aforementioned steps is not performed accurately. To address this issue, the inversion procedure can be modified to yield the APE directly from the experimental isotherm. Considering that the isotherm of Eq. 1 for a single temperature is given by a matrix representation

7 $$\begin{aligned} \textbf{C}_{m \times n} \textbf{V}_{n \times 1} = \textbf{Q}_{m \times 1} \end{aligned}$$

where *m* represents the number of experimental data points for the adsorbed quantity related to the solution concentration, while *n* denotes the order of the virial expansion. The $\textbf{C}_{m \times n}$ matrix of concentrations is given by

$$\begin{aligned} \textbf{C}_{m \times n} = \begin{bmatrix} c_1 &  c_1^2 &  \cdots &  c_1^n \\ c_2 &  c_2^2 &  \cdots &  c_2^n \\ \vdots &  \vdots &  \ddots &  \vdots \\ c_m &  c_m^2 &  \cdots &  c_m^n \end{bmatrix} \end{aligned}$$

the matrix of virial coeficients $\textbf{V}_{n \times 1}$ is represented by

$$\begin{aligned} \textbf{V}_{n \times 1} = \begin{bmatrix} B_1(T) \\ C_2(T) \\ \vdots \\ Z_n(T) \end{bmatrix} \end{aligned}$$

and the vector of adsorbed quantity is given by $\textbf{Q}_{m \times 1}$.

In other words, Eq. 7 can be represented as a tensor operation of order two, where the two variables, *c* and *q*, are correlated in the model given by Eq. 1. Most optimization problems in adsorption can be represented using this scheme, as the fitting procedure is performed separately for each temperature. A third variable can be included in Eq. 7, allowing the problem to be represented as a tensor contraction of order three. Consequently, this approach can be generalized to multiple temperatures. This enables the inversion to be performed using adsorption data across different temperatures simultaneously, where the APE can be retrieved directly from the adsorption quantity without requiring separate determination of the SVC for each temperature. Therefore, the problem can be represented as a tensor contraction along the dimension of the polynomial order *n*

8 $$\begin{aligned} \textbf{C}_{m \times n \times p} \textbf{V}_{n \times 1 \times p} = \textbf{Q}_{m \times 1 \times p} \end{aligned}$$

This representation can be considered a multilinear system and can alternatively be written as an Einstein product [28, 29].

9 $$\begin{aligned} Q_{ik} = \sum _{j=1}^{n} C_{ijk} V_{jk} \end{aligned}$$

As an example, the tensor $\textbf{V}_{n \times 1 \times p}$ for a third-order polynomial expansion is

10 $$\begin{aligned} \begin{aligned} \textbf{V}_{:,:,1}&= \begin{bmatrix} B_1(T_1,E_p) \\ B_1(T_2,E_p) \\ \vdots \\ B_1(T_n,E_p) \end{bmatrix}, \quad \textbf{V}_{:,:,2} = \begin{bmatrix} C_2(T_1) \\ C_2(T_2) \\ \vdots \\ C_2(T_n) \end{bmatrix}, \\ \textbf{V}_{:,:,3}&= \begin{bmatrix} D_3(T_1) \\ D_3(T_2) \\ \vdots \\ D_3(T_n) \end{bmatrix} \end{aligned} \end{aligned}$$

It is important to emphasize that the $B(T_n,E_p)$ terms are not independently fitted, but are instead indirectly determined via inversion of the potential parameters. Since the parameters *A* and $d_0$ cannot be determined separately, they are calculated as a combined quantity $Ad_0$. [26]. Therefore, the calculation is performed knowing the parameters of the APE for a single particle with the surface and the higher-order coefficients $C_2(T)$, $D_3(T)$, and so on.

Lastly, the inverse problem is solved using the Moore–Penrose pseudoinverse to obtain $\textbf{V}_{n \times 1 \times p}$,

11 $$\begin{aligned} \textbf{V}^{(k)} = \left[ \left( \textbf{C}^{(k)}\right) ^T \textbf{C}^{(k)} \right] ^{-1} \left( \textbf{C}^{(k)}\right) ^T \textbf{Q}^{(k)} \end{aligned}$$

The equation above is coupled with the Levenberg–Marquardt algorithm to determine the nonlinear parameters of the APE. This multilinear approach for solving the inverse problem is referred to as Model 3.

## Results and discussion

### Adsorption datasets and inversion strategy

Since the second virial coefficient corresponds to the linear intercept, the isotherm data must exhibit near-linear behavior at low concentrations. Therefore, the experimental dataset should include a sufficient number of points in this concentration range at different temperatures. To validate the methodology, experimental datasets exhibiting these characteristics were selected for adsorption systems in both aqueous and gas phases. For adsorption in aqueous solution, two datasets were selected for validation of the proposed methodology. The first dataset was obtained from the work of Oueslati et al., in which the adsorption of Reactive Red 120 dye (RR 120) on activated carbon (AC) was investigated [30]. The second dataset is avaliable on Calvete et al. works and refers to the adsorpiton of Procion Red MX 3Bdye (PR-3B) on AC. These datasets provide extensive coverage of low-concentration measurements exhibiting near-linear behavior. Additionally, the study reports adsorption isotherms measured at six different temperatures, making this dataset particularly suitable for evaluating the efficiency of the model. For the selection of gas-phase adsorption data, the same criteria were applied, and the dataset reported by Sghaier et al. on CO$_2$ adsorption over AC was selected. [32]. In both cases, the experimental data available in graphical form were extracted using WebPlotDigitizer software [33].

The Lennard–Jones 10-4 potential, given by Eq. 5, was selected for the inversion procedure because it is widely employed to describe adsorbate–surface interactions and requires only a small number of adjustable parameters. However, the inversion strategy proposed in this work is not restricted to a specific potential model. Other adsorption potentials, such as the Steele potential [34], can also be incorporated into the same framework without modification of the underlying methodology.

**Fig. 1:**
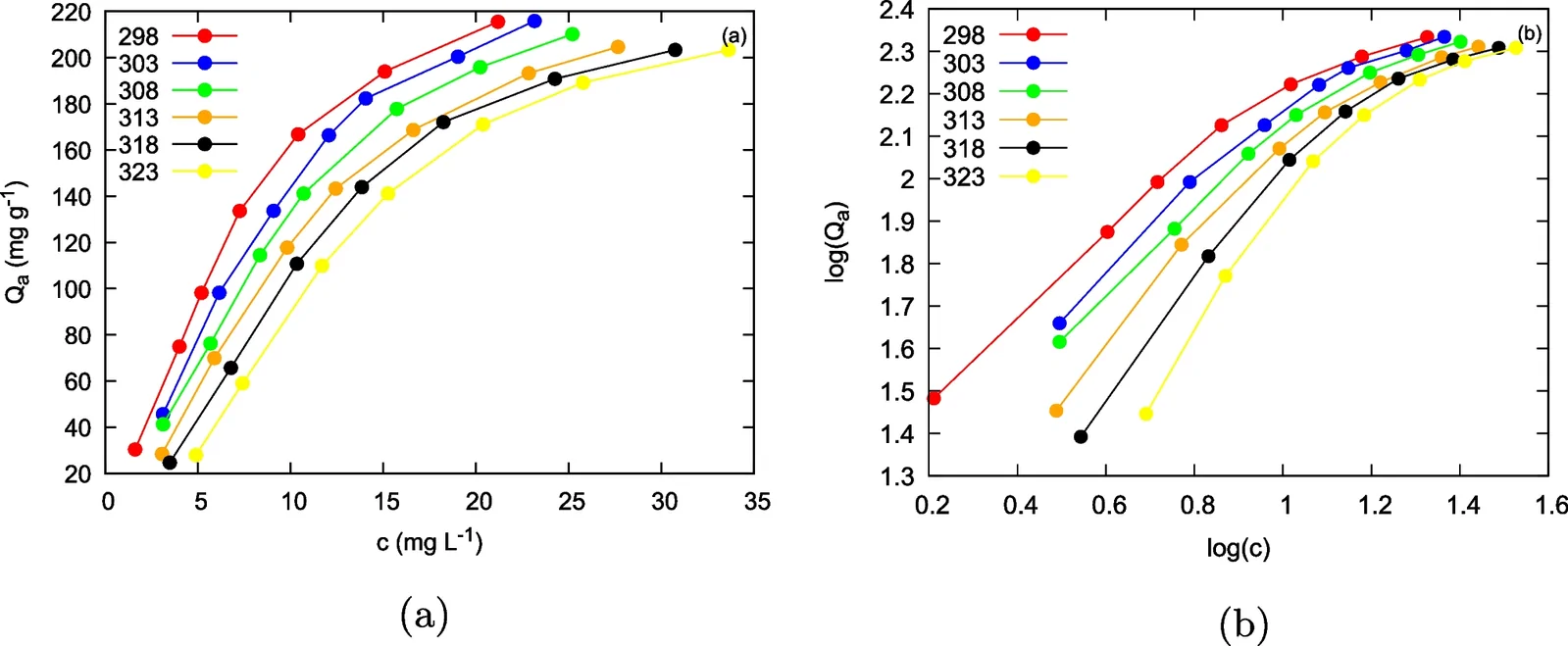
**a** Experimental isotherm for RR120 on AC data extracted from the work of Oueslati et al. [30]. **b** Logarithmic transformation used to evaluate the linearity range

### Inversion of the APE for RR120 adsorption on AC

The data extracted from Oueslati et al. are presented in Fig. 1a and can be characterized as an ideal dilute solution behavior, as evidenced by the linear relationship between logarithmic concentration and adsorption quantity, Fig. 1b. This linear regime, maintained up to $\log \, C \approx 1.2$, provides an optimal range for model validation, while data beyond this threshold were excluded due to deviations from ideality that could compromise the inversion procedure.

As can be seen in Fig. 1a, the adsorbed quantity is not zero when the concentration is zero. This deviation is particularly noticeable at higher isotherm temperatures, particularly at 323 K. Consequently, the estimation of $B_1$ may be inaccurate, since the limit defined in Eq. 2 does not yield the correct value of the thermodynamic property. This issue can be addressed by adding a coefficient indendent of *c* to Eq. 1, following a procedure similar to baseline correction [35] or an isotherm model proposed by Gupta and Gosal [10]. Accordingly, the isotherm can be expressed by adding the $A_0$ coefficient as:

12 $$\begin{aligned} Q_a=A_0(\mu ,T) + B_1(\mu ,T)c+C_2(\mu ,T)c^2+D_3(\mu ,T)c^3+... \end{aligned}$$

For a fair comparison among the different models employed in this work, Eq. 6 used in Model 1 was modified by including an independent term $A_0$. It is important to emphasize that this parameter does not have a direct physical meaning. Instead, it serves as a correction term to account for deviations in the experimental data as *c* approaches zero.

To evaluate the quality of both the isotherm fitting and the inversion procedure, the adjusted coefficient of determination $R^2$ and the adjusted root mean square deviation (RMSD) were used as performance metrics [36]

13 $$\begin{aligned} R^2= \left[ 1- \frac{ \sum _{i=1}^{n} \left( Q_{i,\textrm{exp}}-Q_{i,\textrm{model}}\right) ^2 }{ \sum _{i=1}^{n} \left( Q_{i,\textrm{exp}}-\overline{Q}_{\textrm{exp}}\right) ^2 } \right] \left( \frac{n-1}{n-p} \right) \end{aligned}$$

14 $$\begin{aligned} \textrm{RMSD} = \sqrt{ \frac{ \sum _{i=1}^{n} \left( Q_{i,\textrm{exp}}-Q_{i,\textrm{cal}}\right) ^2 }{ n-p } } \end{aligned}$$

Being $Q_{i,\textrm{exp}}$ and $Q_{i,\textrm{model}}$ the experimental adsorbed quantity and the value calculated by the model, respectively. These metrics offer an advantage over traditional measures because they penalize models with a larger number of parameters *p*.

To invert the potential energy using Model 1, the second adsorption virial coefficient was first obtained by fitting Eq. 6. In the absence of the $A_0$ correction, the model isotherm did not adequately reproduce the experimental data, as evidenced by the global $R^2$ and RMSD values across all temperatures.

Despite the poor isotherm fit, the inversion yielded potential parameters successfully. Since the inversion procedure was performed in two steps, an additional test was conducted to evaluate the reliability of the approach. Using the inverted parameters, *B*(*T*) was recalculated, and the isotherms were subsequently recalculated using the remaining parameters adjusted in Eq. 6. Analysis of the individual $R^2$ and *RMSD* values at each temperature shows that the properties obtained from the inversion procedure do not adequately reproduce the experimental data, as shown in Tables 1 and 3.

With the inclusion of the independent term $A_0$ in Model 1, the inversion procedure improves considerably, as the isotherm fitting is significantly enhanced, as evidenced by the improved $R^2$ and RMSD values. In this case, inversion of the potential through *B*(*T*) yields excellent results. However, when the isotherms are recalculated using the inverted potential, the agreement is not fully satisfactory, with $R^2 = 0.954$. By comparison, isotherm fitting using models based on a statistical mechanics approach provides significantly better agreement, with $R^2 > 0.98$ [37–39].

Using Model 2 with Eqs. 1 and 12, the virial coefficients were obtained considering both second- and third-order expansions. Both models reproduce the experimental data within the linearity limit. Moreover, the potential parameters derived from the adjusted virial coefficient $B_1$ show good agreement with the virial data. To evaluate the quality of the procedure, the second virial coefficient must be calculated from the potential energy, and the adsorbed quantity subsequently estimated using the higher-order virial coefficients obtained by fitting Eqs. 1 and 12. Despite the high $R^2$ values obtained during the fitting steps, the inverted potential does not accurately reproduce the experimental data, as indicated by the statistic metrics of the inverted potential. Additionally, the inclusion of the $A_0$ term does not improve the fitting performance, nor does the inclusion of higher-order terms in the virial expansion. Although higher-order expansions are expected to yield better results in the inversion process, this approach does not provide satisfactory outcomes.

**Table 1:** Statistical metrics for the different steps (isotherm fitting, potential inversion, and recalculated isotherms) in the inversion of the APE for RR120 adsorption on AC. Note that Model 3 reports only the recalculated isotherms, since the inversion is performed in a single step

Model	Polynomial order	$A_0$ correction	Isotherm fitting		Potential inversion		Recalculated isotherm	
			$R^2$	RMSD	$R^2$	RMSD	$R^2$	RMSD
1	n/a	Absent	0.6572	35.7810	0.9991	0.0612	0.7059	32.6633
		Present	0.9460	14.2016	0.9974	0.2827	0.9549	12.6931
2	2nd	Absent	0.9833	7.8990	0.9983	0.1817	0.9836	7.0458
		Present	0.9953	4.2051	0.9505	0.6007	0.9716	9.5222
2	3rd	Absent	0.9867	7.0351	0.9896	0.6153	0.9588	10.6935
		Present	0.9980	2.7390	0.8899	1.2406	0.8557	23.0768
3	2nd	Absent	n/a	n/a	n/a	n/a	0.9819	7.7720
		Present	n/a	n/a	n/a	n/a	0.9957	3.5650
3	3rd	Absent	n/a	n/a	n/a	n/a	0.9851	7.1448
		Present	n/a	n/a	n/a	n/a	0.9982	2.2551

**Fig. 2:**
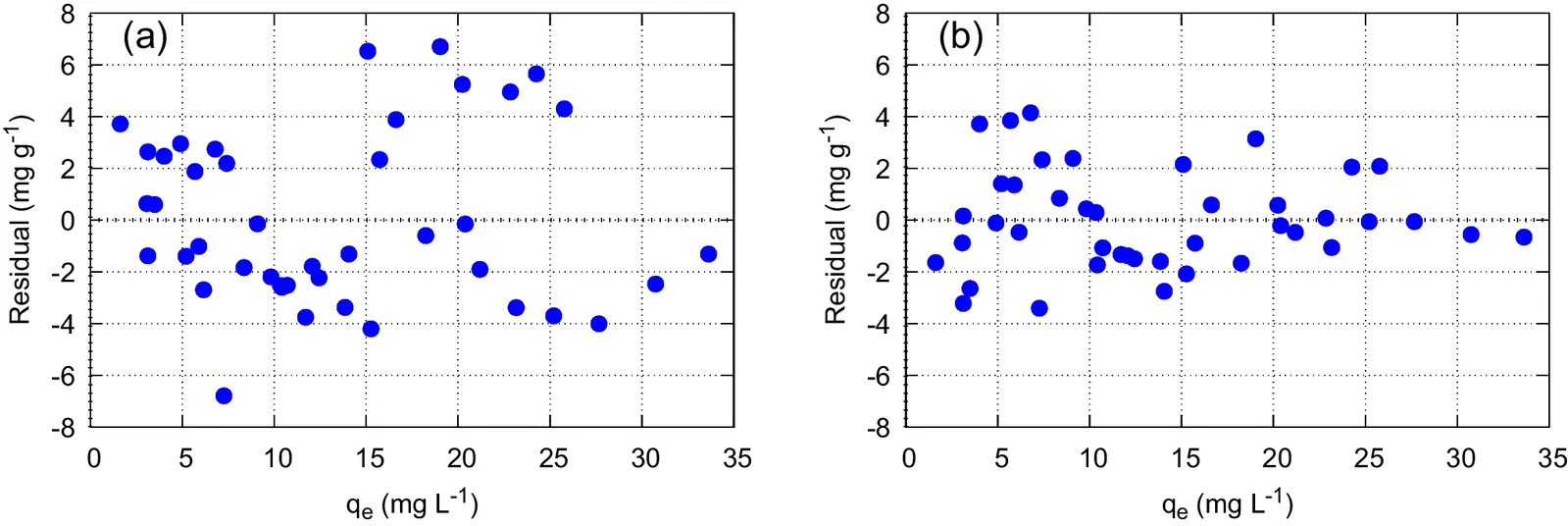
Residual analysis for the APE inversion for RR120 on AC using Model 3 with $A_0$ correction: **a** second-order and **b** third-order expansions

As can be seen, independent determination of *B*(*T*) followed by potential inversion does not yield parameters capable of adequately describing the experimental data, due to the ill-posed nature of the problem. This arises from error propagation when the inversion is performed in separate steps, introducing significant instability into the procedure. This issue is overcome by directly inverting the APE from adsorption data, as proposed here through Model 3. The enhanced stability of Model 3 can be attributed to the coupled treatment of the virial coefficients and APE parameters. By determining both quantities simultaneously, the inversion avoids the accumulation of errors associated with intermediate calculations. This characteristic reduces the sensitivity of the solution to uncertainties in the experimental data and improves the robustness of the inversion procedure.

As shown in Table 1, this approach provides excellent results and stabilizes the solution of the ill-posed problem, particularly when the $A_0$ correction is included, yielding highly accurate descriptions of the experimental isotherms. For Model 3, the columns corresponding separately to isotherm fitting and potential inversion are not reported, since the inversion is performed in a single step. The metrics reported for the recalculated isotherms correspond to the values obtained after inversion APE parameters together with the remaining model parameters.

**Table 2:** Fitted parameters of Model 3 for the RR120 adsorption on AC. The parameter $B_1$ was calculated from the inverted APE parameters solving Eq. 4, with values given by $\epsilon /kT = {1.689}$ and $A_d = 0.4067$ g$^{-1}$ L

Temperature (K)	$A_0$ (mg g$^{-1}$)	$B_1$ (mg g$^{-1}$ mg $^{-1}$ L)	$C_2$ (mg g$^{-1}$ mg $^{-2}$ L$^{2}$)	$D_3$ (mg g$^{-1}$ mg $^{-3}$ L$^{3}$)
298	−11.9492	26.7688	−1.1133	0.0168
303	−22.8695	24.6297	−0.8932	0.0118
308	−25.6906	22.7248	−0.7847	0.0101
313	−31.0288	21.0227	−0.6720	0.0079
318	−39.9372	19.4968	−0.5231	0.0047
323	−51.4967	18.1249	−0.4152	0.0030

**Fig. 3:**
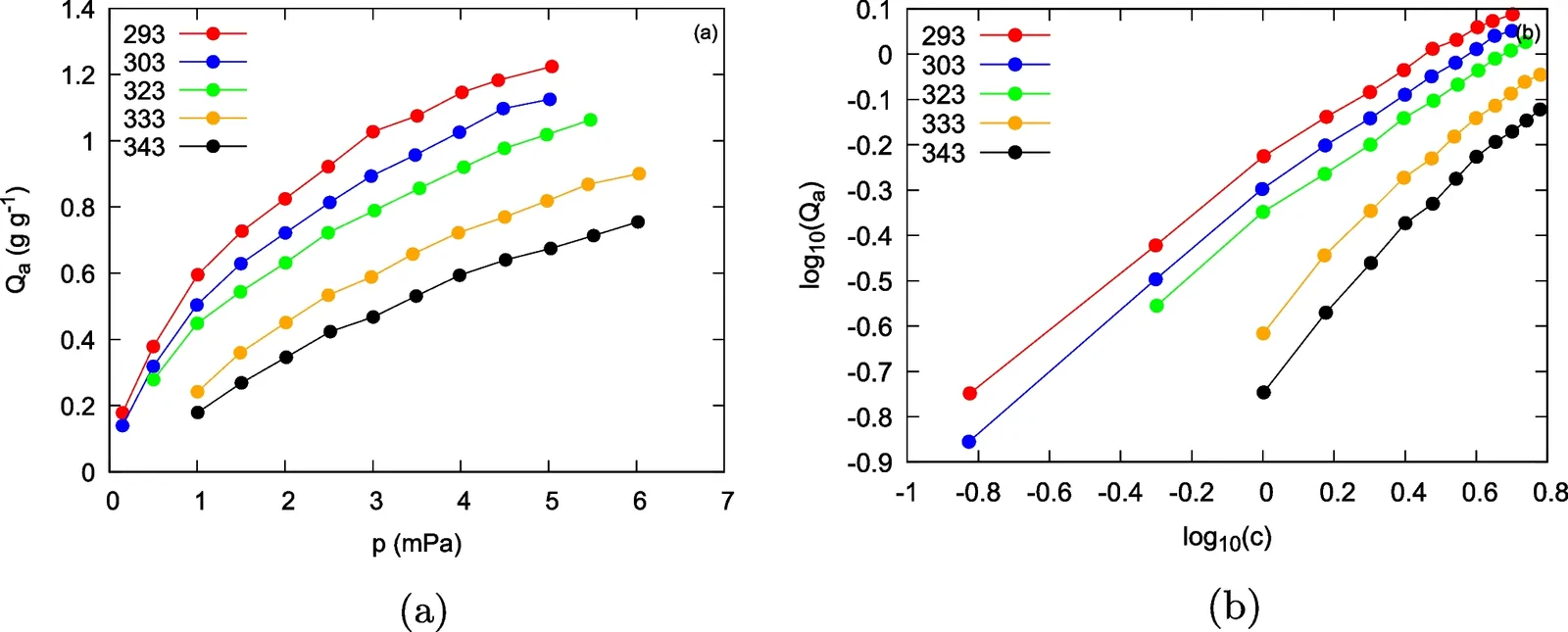
**a** Experimental isotherm for CO$_2$ on AC data extracted from the work of Sghaier et al. [32]. **b** Logarithmic transformation used to evaluate the linearity range

The $R^2$ values for the second- and third-order polynomial expansions are 0.996 and 0.998, respectively, comparable to those of the best isotherm models. Despite the increased number of parameters introduced by the higher polynomial order and the $A_0$ term, which penalizes the statistical metrics, $R^2$ increases and *RMSD* decreases. These results indicate that Model 3 with the $A_0$ correction provides the most effective inversion procedure. Moreover, a clearer trend is observed when Model 3 is employed, with the fitting error decreasing due first to the $A_0$ correction and subsequently to the increase in polynomial order, as expected from dilute solution theory. This trend is not observed for Models 1 and 2, providing further evidence that these models do not recover the potential energy reliably from experimental isotherm data.

Considering that the best inversion results were obtained with Model 3 including the $A_0$ correction, and that the $R^2$ and *RMSD* values for the second- and third-order expansions are relatively similar, a deeper analysis was carried out. Residual analysis for both expansions was performed, as shown in Fig. 2. The residual analysis confirms that the third-order expansion provides a better inversion result, as it exhibits smaller residual amplitudes than the second-order expansion, particularly in the intermediate concentration range. These results indicate that the APE for the adsorption of RR120 dye on AC is best described using Model 3 with the $A_0$ correction and a third-order expansion. The parameters obtained from the inversion are presented in Table 2.

### inversion of the APE for CO$_2$ adsorption on AC

To evaluate the performance of the different models in the inversion of the APE for CO$_2$ adsorption on AC, the same analysis employed for RR120 adsorption was applied. The experimental data and the logarithmic transformation are shown in Fig. 3. For this dataset, the behavior is close to linear over the entire concentration range; therefore, all data points were used in the inversion procedure. The results obtained with the different models are presented in Table 3.

**Table 3:** Statistical metrics for the different steps (isotherm fitting, potential inversion, and recalculated isotherms) in the inversion of the APE for CO$_2$ adsorption on AC. Note that Model 3 reports only the recalculated isotherms, since the inversion is performed in a single step

Model	Polynomial order	$A_0$ correction	Isotherm fitting		Potential inversion		Recalculated isotherm	
			$R^2$	RMSD	$R^2$	RMSD	$R^2$	RMSD
1	n/a	Absent	0.9982	0.0121	0.8671	0.2003	0.8170	0.1215
		Present	0.9983	0.0119	0.9372	0.1556	0.7888	0.1312
2	2nd	Absent	0.9654	0.0531	0.9194	0.0475	0.7317	0.1398
		Present	0.9936	0.0229	0.9974	0.0053	0.9904	0.0217
2	3rd	Absent	0.9897	0.0290	0.9375	0.0649	0.5158	0.1970
		Present	0.9975	0.0143	0.9912	0.0153	0.9691	0.0483
3	2nd	Absent	n/a	n/a	n/a	n/a	0.9538	0.0540
		Present	n/a	n/a	n/a	n/a	0.9939	0.0216
3	3rd	Absent	n/a	n/a	n/a	n/a	0.9824	0.0327
		Present	n/a	n/a	n/a	n/a	0.9976	0.0129

**Fig. 4:**
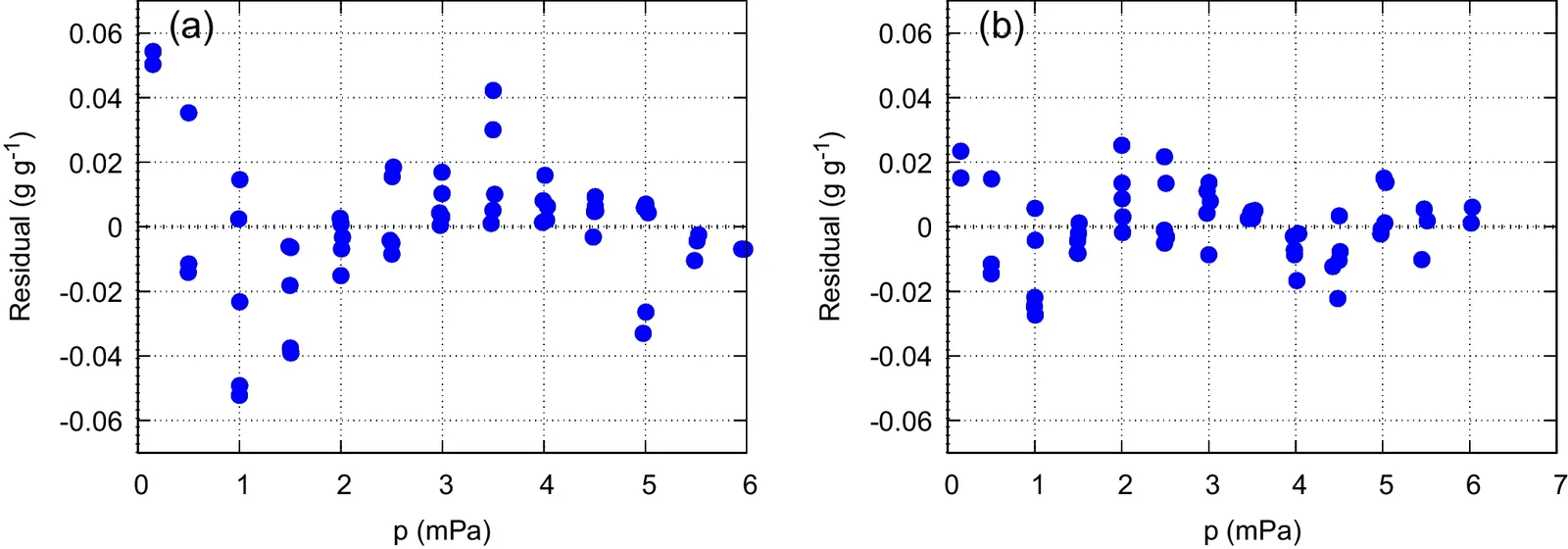
Residual analysis for the APE inversion for CO$_2$ on AC using Model 3 with $A_0$ correction: **a** second-order and **b** third-order expansions

Similar to the inversion results for the APE of RR120 adsorption on AC, Models 1 and 2 do not exhibit a consistent trend in the statistical metrics due to the instability arising from the ill-posed nature of the problem. In Model 1, inclusion of the term $A_0$ deteriorates the inversion results, whereas in Model 2 it significantly improves inversion performance. However, increasing the polynomial order in Model 2 hinders the inversion results. For example, in Model 2 without $A_0$ correction, instability becomes evident during the inversion procedure, leading to substantial degradation of the statistical metrics. In particular, the $R^2$ value decreases from approximately 0.9 to below 0.7 in the recalculated isotherm. These results provide clear evidence of the ill-posed nature of the problem and the impact of error propagation in the inversion process. This instability is mitigated when APE is inverted using Model 3. The importance of the $A_0$ correction is again evident. Although this dataset does not visually exhibit a clear issue in the p→0 limit, satisfying the correct Henry’s law behavior in this region remains essential, highlighting the role of the $A_0$ parameter.

For CO$_2$ adsorption on AC, Model 3 clearly overcomes the ill-posed nature of the problem and follows the consistent trends expected from statistical mechanics theory. The second- and third-order expansions provide the most satisfactory results. To further assess inversion quality, a residual analysis was performed, and the results are shown in Fig. 4. The third-order expansion shows smaller residual amplitudes, particularly in the low-pressure region, which is the most critical range for correctly describing Henry’s law behavior. The parameters obtained with Model 3, including the $A_0$ correction and third-order expansion, are presented in Table 4.

### Inversion of the APE for PR-3B adsorption on AC

**Fig. 5:**
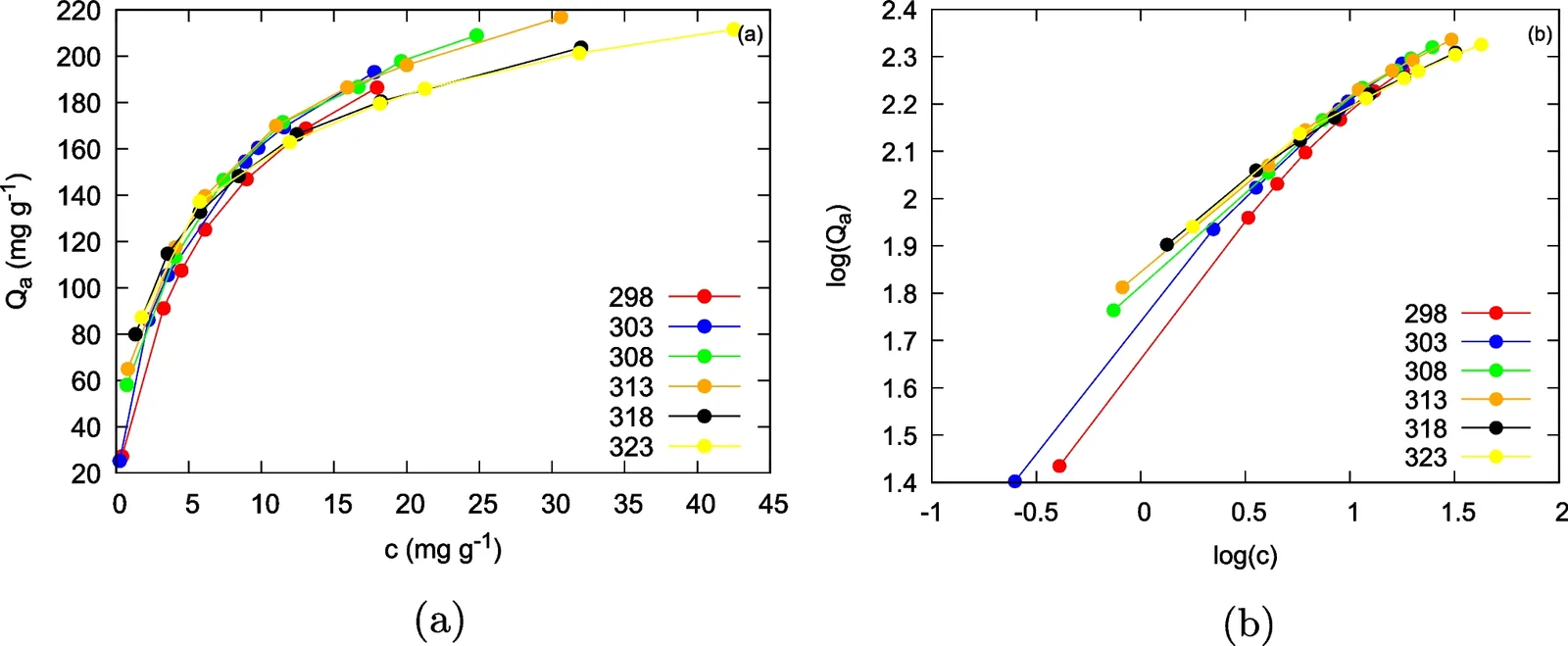
**a** Experimental isotherm for PR-3B on AC data extracted from the work of Calvete et al. [31]. **b** Logarithmic transformation used to evaluate the linearity range

Similar to the other datasets, the same inversion procedure was applied to the adsorption data of PR-3B onto AC. As shown in the isotherms presented in Fig. 5, this system exhibits a more complex temperature dependence than the previously analyzed datasets. For the latter, the isotherm slopes and adsorption capacities display a clear and consistent dependence on temperature, with the adsorption capacity decreasing as the temperature increases. In contrast, the PR-3B isotherms do not exhibit a clear monotonic trend in either slope or adsorption capacity across the investigated temperatures. Although this behavior makes the system more difficult to interpret, it also provides a more stringent test of the proposed methodology, since the inversion procedure must account for experimental data exhibiting a less regular temperature dependence.

**Table 4:** Fitted parameters of Model 3 for the CO$_2$ adsorption on AC. The parameter $B_1$ was calculated from the inverted APE parameter solving Eq. 4, with values given by $\epsilon /kT={1.788}$ and $A_d=0.0057$ g g$^{-1}$ mPa$^{-2}$

Temperature (K)	$A_0$ (g g$^{-1}$)	$B_1$ (g g$^{-1}$ mPa$^{-1}$)	$C_2$ (g g$^{-1}$ mPa$^{-2}$)	$D_3$ (g g$^{-1}$ mPa$^{-3}$)
293	0.1183	0.5567	−0.1132	0.0093
303	0.0968	0.4649	−0.0893	0.0077
323	0.1359	0.3356	−0.0480	0.0032
333	−0.0118	0.2895	−0.0343	0.0019
343	−0.0482	0.2519	−0.0306	0.0018

The results obtained from the inversion using Models 1, 2, and 3 are presented in Table 5. The irregular temperature dependence observed in this dataset significantly affects the performance of Models 1 and 2. In all tested configurations, these models yield $R^2 < 0.9$, and under some conditions, negative $R^2$ values are obtained. A negative $R^2$ indicates that the isotherms recalculated from the inverted adsorption potential energy (APE) parameters provide a poorer description of the experimental data than a model based simply on the mean value of the observations. Furthermore, no clear improvement in the inversion results is observed with increasing polynomial order or with the inclusion of the $A_0$ correction term.

**Table 5:** Statistical metrics for the different steps (isotherm fitting, potential inversion, and recalculated isotherms) in the inversion of the APE for PR-3B adsorption on AC. Model 3 reports only the recalculated isotherms, since the inversion is performed in a single step

Model	Polynomial order	$A_0$ correction	Isotherm fitting		Potential inversion		Recalculated isotherm	
			$R^2$	RMSD	$R^2$	RMSD	$R^2$	RMSD
1	n/a	Absent	−0.8963	69.1202	0.6198	12.0349	−12.7301	185.5684
		Present	0.9983	0.0119	0.9372	0.1556	0.7888	0.1312
2	2nd	Absent	0.5767	32.6564	0.8493	1.7594	0.3566	31.3459
		Present	0.9594	10.1142	0.9410	1.3671	0.8682	15.6736
2	3rd	Absent	0.9897	0.0290	0.9375	0.0649	0.5158	0.1970
		Present	0.9924	4.3851	0.7578	4.3327	−0.1522	53.7068
3	2nd	Absent	n/a	n/a	n/a	n/a	0.6056	29.7676
		Present	n/a	n/a	n/a	n/a	0.9629	8.9365
3	3rd	Absent	n/a	n/a	n/a	n/a	0.8362	18.6668
		Present	n/a	n/a	n/a	n/a	0.9900	4.1481

**Fig. 6:**
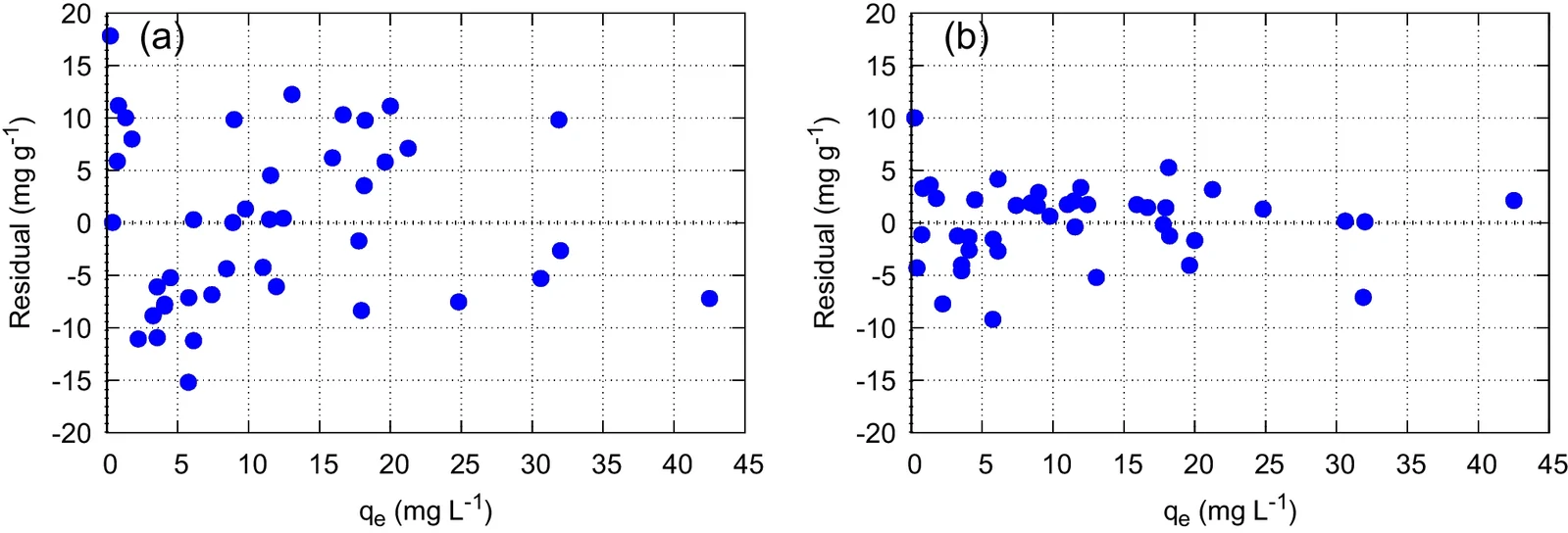
Residual analysis for PR-3B adsorption on AC using Model 3 with $A_0$ correction: **a** second-order and **b** third-order expansions

In contrast, Model 3 provides substantially better results, despite the increased complexity and irregular temperature dependence of this dataset. Although its performance is somewhat inferior to that obtained for the previously analyzed datasets, the inversion remains highly satisfactory. In particular, the third-order expansion with the $A_0$ correction yields an $R^2$ value of approximately 0.99, indicating an excellent agreement between the experimentally measured and recalculated isotherms. This result is particularly relevant because it demonstrates that Model 3 is capable of providing a reliable inversion even for a dataset exhibiting a less regular temperature dependence, thereby providing additional evidence of the robustness and general applicability of the proposed methodology.

The residual analysis comparing the performance of Model 3 with $A_0$ correction at the 2nd- and 3rd-order expansions is presented in Fig. 6. As can be seen, the 2nd-order inversion exhibits larger deviations at low concentrations, with the residuals being predominantly negative and not evenly distributed around zero. This behavior is improved in the 3rd-order inversion, for which the residuals are smaller at low concentrations and more evenly distributed around zero. These results indicate that the 3rd-order expansion provides an improved description of the experimental data, particularly in the low-concentration regime. The APE inversion parameters and the fitted virial coefficients obtained under the best inversion conditions for this dataset, using Model 3 with the $A_0$ correction and a third-order expansion, are presented in Table 6.

### General trends of the virial coefficients

Regarding the virial coefficients obtained for both systems (RR120 and CO$_2$ adsorption), the observed temperature dependence reflects the balance between intermolecular interactions and thermal effects. A decrease in the value of $B_1$ (SVC) with increasing temperature is observed, which occurs due to the reduction in the adsorption energy. In particular, for the adsorption of RR120, which occurs in solution, the interactions between the adsorbate and the surface are more favorable than those between the dye molecules and the solvent.

The negative values of $C_2$ indicate that adsorption is dominated by repulsive interactions or competition for adsorption sites. This effect is more pronounced at lower temperatures, where reduced thermal motion enhances the influence of intermolecular interactions. As the temperature increases, thermal agitation weakens these interactions, leading to less negative values and indicating a shift toward entropy-dominated behavior. The stronger negativity observed in the aqueous phase suggests that additional factors such as solvation and steric constraints further enhance competitive effects compared to the gas phase.

In contrast, the slightly positive values for $D_3$ indicate that many-body correlations contribute cooperatively to adsorption. These correlations are more significant at lower temperatures, where structured arrangements of adsorbed molecules are more stable. As temperature increases, thermal motion disrupts these arrangements, reducing the magnitude of the third virial coefficient and indicating a diminished role of higher-order interactions.

**Table 6:** Fitted parameters of Model 3 for PR-3B adsorption on AC. The parameter $B_1$ was calculated from the inverted APE parameter by solving Eq. 4, with values given by ϵ/kT=3.610 and $A_d=0.0012$ g$^{-1}$ L

Temperature (K)	$A_0$ (mg g$^{-1}$)	$B_1$ (mg g$^{-1}$ mg$^{-1}$ L$^{1}$)	$C_2$ (mg g$^{-1}$ mg$^{-2}$ L$^{2}$)	$D_3$ (mg g$^{-1}$ mg$^{-3}$ L$^{3}$)
298	10.5888	31.1054	−2.2931	0.0618
303	28.9327	25.7174	−1.6404	0.0401
308	41.6561	21.3985	−1.0999	0.0206
313	54.1086	17.9130	−0.7963	0.0126
318	64.5812	15.0820	−0.6771	0.0107
323	68.2714	12.7684	−0.4468	0.0053

**Table 7:** Mean values and standard deviations obtained from Model 3 with the addition of artificial errors to the adsorption isotherms of R120 on AC

Error	ϵ (kT)	*Ad* (mg g$^{-1}$)	$R^2$	*RMSD*
1 %	1.64±0.20	0.55±0.33	0.9982±0.0003	2.11±0.23
5 %	1.90±0.71	0.87±1.15	0.9885±0.0042	5.05±1.07
10 %	1.96±1.13	1.47±1.45	0.9629±0.0132	9.41±2.09

### Sensitivity and robustness of model 3

To evaluate the sensitivity and robustness of Model 3, a series of analyses was performed. The adsorption dataset of Reactive Red 120 (RR120) on activated carbon (AC) was selected for this analysis because it contains the largest number of experimental data points among the datasets considered, providing a more rigorous test of the method. Furthermore, all calculations were carried out using the third-order expansion with the $(A_0)$ correction, as this configuration yielded the most accurate results in the previous analyses.

The first assessment examined the dependence of the inversion procedure on the initial estimates of the nonlinear parameters, (ϵ) and $(A_d)$. Since the remaining parameters enter the model linearly, they were determined directly using the Moore–Penrose pseudoinverse. A set of randomly generated initial guesses for (ϵ), ranging from 0.1 to 10 (kT), and $(A_d)$, ranging from 0.01 to 1 (g$^{-1}$ L), was employed, and the inversion procedure was repeated for each condition.

The sensitivity analysis showed that the inversion results were remarkably insensitive to the initial estimates, since the standard deviation of ϵ/kT and $A_d$ was only $9.93\times 10^{-8}$ and $1.31\times 10^{-6}$, respectively. These extremely small deviations demonstrate that the inversion converges to essentially the same solution over a broad range of initial estimates, indicating a very low sensitivity of the obtained APE parameters to the initial guesses.

The robustness of Model 3 was further assessed by introducing controlled artificial noise into the experimental isotherm data for the adsorption of R120 onto AC. Normally distributed random errors corresponding to 1, 5, and 10 % of the experimental values were independently added to the original dataset. For each noise level, 100 perturbed isotherms were generated using randomly sampled errors, and the inversion procedure was subsequently performed for each realization. The mean values of the estimated parameters ϵ and *Ad*, together with their corresponding standard deviations, are presented in Table 7.

As can be seen, the parameter ϵ, which represents the depth of the adsorption potential well, exhibits a high degree of stability with respect to the introduction of artificial noise. Its mean values remain close to the value obtained from the original experimental dataset, while the corresponding standard deviations remain relatively small even at the highest noise level considered. This behavior indicates that the estimation of the potential well depth is not significantly affected by moderate perturbations of the experimental data.

In contrast, the parameter *Ad* exhibits a substantially larger dispersion as the noise level increases. This behavior is expected because *Ad* is defined as the product of two quantities, namely the surface area *A* and the minimum potential coordinate *d*. Consequently, variations in the experimental data can be propagated through this product, resulting in greater variability in the estimated value of *Ad*. This behavior is also consistent with the inherent characteristics of ill-posed inverse problems, for which different combinations of model parameters may provide similarly satisfactory descriptions of the available data. Therefore, the relatively large standard deviations observed for *Ad* should not necessarily be interpreted as a failure of the inversion procedure, but rather as an indication of the greater sensitivity of this composite parameter to perturbations in the input data.

Despite this sensitivity, the overall quality of the inversions remains high, as indicated by the $R^2$ and RMSD values obtained for the perturbed datasets. Even when artificial errors of up to 10 % were introduced, the inversion procedure continued to provide satisfactory fits to the resulting isotherms. This result demonstrates that Model 3 maintains its predictive capability under controlled perturbations of the experimental data. It is worth noting that the original experimental measurements already contain an intrinsic level of experimental uncertainty; therefore, the successful recovery of the adsorption parameters after the deliberate introduction of additional random errors provides further evidence of the numerical robustness of the proposed inversion procedure. If the model were highly unstable with respect to perturbations in the input data or strongly dependent on the particular realization of the experimental errors, substantial deterioration of the $R^2$ and RMSD values would be expected as the noise level increased. The absence of such a pronounced deterioration supports the robustness of Model 3 against experimental data perturbations.

To further assess the numerical stability of the inversion procedure, the condition number of the concentration matrix involved in the Moore-Penrose pseudoinverse was evaluated for each temperature. The condition number was calculated as $\kappa (\textbf{C}^{(k)})=\sigma _{\max }/\sigma _{\min }$, where $\sigma _{\max }$ and $\sigma _{\min }$ are the largest and smallest singular values of $\textbf{C}^{(k)}$, respectively [40]. For the RR120/AC dataset with model 3, the condition numbers ranged from $6.79\times 10^{3}$ to $3.33\times 10^{4}$ across the investigated temperatures. These values indicate a moderately conditioned inversion problem, with the matrices remaining sufficiently far from numerical singularity. Therefore, although the polynomial structure of the concentration matrix introduces a certain degree of conditioning, the Moore–Penrose pseudoinverse does not lead to severe numerical instability for the dataset investigated. This result is consistent with the sensitivity and noise analyses presented above, which showed that the recovered parameters remain stable under changes in the initial estimates and perturbations of the experimental data.

## Conclusion

In this work, a new methodology is proposed to determine the APE directly from adsorption isotherm data, based on Henry’s law adsorption. The traditional approach involves a two-step procedure, in which the SVC is first obtained from the isotherm data and subsequently used for inversion of the APE. However, due to the ill-posed nature of the problem, this approach introduces significant instability and error propagation during the inversion process.

The proposed approach, referred as Model 3, formulates the problem as a tensorial contraction and was evaluated using three experimental datasets: adsorption of RR120 and PR-3B dyes on AC in aqueous solution and CO$_2$ adsorption on AC in the gas phase. In all cases, increasing the polynomial order and including the baseline term $A_0$ improved the inversion results. Although these modifications increase the number of model parameters, the use of adjusted $R^2$ and *RMSD*, which penalize model complexity, confirms the improvement. Under these conditions, the method achieves $R^2 \sim 0.99$, consistent with the best-performing adsorption models. In contrast, Models 1 and 2, which rely on a two-step procedure, do not exhibit a consistent trend and, in some cases, yield inversion results with $R^2 < 0.7$, highlighting the inherent instability of the approach.Additionally, Model 3 demonstrated robust performance in the presence of artificially introduced experimental errors and remarkable stability with respect to different initial estimates of the nonlinear parameters, supporting the reliability of the proposed inversion procedure.

Overall, the results suggest that direct inversion of the APE within the isotherm fitting framework provides a more stable strategy for treating the inverse problem under the conditions investigated. By avoiding intermediate steps and reducing error propagation, the proposed methodology allows a consistent description of adsorption behavior for the studied systems. Nevertheless, the applicability of the approach depends on the availability of experimental adsorption isotherms that obey Henry’s law and contain a sufficient number of experimental points in the linear low-concentration region at different temperatures. Under these conditions, the methodology represents a promising framework for the determination of adsorption potential energies directly from experimental isotherm data.

## Data Availability

The datasets generated during this work are available from the corresponding author on reasonable request.

## References

[1] Katheresan V, Kansedo J, Lau SY (2018) Efficiency of various recent wastewater dye removal methods: a review. J Environ Chem Eng 6(4):4676–4697. 10.1016/j.jece.2018.06.060

[2] De Gisi S, Lofrano G, Grassi M, Notarnicola M (2016) Characteristics and adsorption capacities of low-cost sorbents for wastewater treatment: a review. Sustain Mater Technol 9:10–40. 10.1016/j.susmat.2016.06.002

[3] Sultana M, Rownok MH, Sabrin M, Rahaman MH, Alam S (2022) A review on experimental chemically modified activated carbon to enhance dye and heavy metals adsorption. Clean Eng Technol 6:100382. 10.1016/j.clet.2021.100382

[4] Tran HN, You SJ, Hosseini-Bandegharaei A, Chao HP (2017) Mistakes and inconsistencies regarding adsorption of contaminants from aqueous solutions: a critical review. Water Res 120:88–116. 10.1016/j.watres.2017.04.014 28478298

[5] Alrefaee SH, Aljohani M, Alkhamis K, Shaaban F, El-Desouky MG, El-Bindary AA, El-Bindary MA (2023) Adsorption and effective removal of organophosphorus pesticides from aqueous solution via novel metal-organic framework: adsorption isotherms, kinetics, and optimization via box-behnken design. J Mol Liq 384:122206. 10.1016/j.molliq.2023.122206

[6] Abid LH, Mussa ZH, Deyab IF, Al-Ameer LR, Al-Saedi HFS, Al-Qaim FF, Kamyab H, Rajendran S, Imran AF, Yaseen ZM (2025) Walnut shell as a bio-activated carbon for elimination of malachite green from its aqueous solution: adsorption isotherms, kinetics and thermodynamic studies. Results Chem 14:102124. 10.1016/j.rechem.2025.102124

[7] Ranjan P, Patra T, Suin S, Narayanasamy S (2019) Inducing dye-selectivity in graphene oxide for cationic dye separation applications. Mater Chem Phys 226:350–355. 10.1016/j.matchemphys.2019.01.047

[8] Erastova V, Degiacomi MT, Fraser DG, Greenwell HC (2017) Mineral surface chemistry control for origin of prebiotic peptides. Nat Commun 8:2033. 10.1038/s41467-017-02248-y PMC5725419 29229963

[9] Guo L, Xiao S, Zhang X (2016) Modeling adsorption with lattice Boltzmann equation. Sci Rep 6(1):27134. 10.1038/srep27134 PMC4891696 27256325

[10] Ghosal PS, Gupta AK (2017) Development of a generalized adsorption isotherm model at solid-liquid interface: a novel approach. J Mol Liq 240:21–24. 10.1016/j.molliq.2017.05.042

[11] Justino DD, Alves MO, Galvão BR, Santamaría R, De Sousa FB, Ortega PF (2023) The effects of functionalization on graphene oxide for organic dye adsorption: an experimental-theoretical study using electronic structure calculations and statistical mechanical modeling. J Mol Liq 387:122612. 10.1016/j.molliq.2023.122612

[12] Lamine AB, Bouazra Y (1997) Application of statistical thermodynamics to the olfaction mechanism. Chem Senses 22(1):67–75. 10.1093/chemse/22.1.67 9056086

[13] Khalfaoui M, Knani S, Hachicha M, Lamine AB (2003) New theoretical expressions for the five adsorption type isotherms classified by BET based on statistical physics treatment. J Colloid Interface Sci 263(2):350–356. 10.1016/S0021-9797(03)00139-5 12909023

[14] Amaral TKM, dos Santos GO, de Oliveira PSC, Pires NJ, de Castro VG, Silva GG, Denadai AML, Alves MO, Trigueiro JPC, Lavall RL, Ortega PFR (2020) The effect of debris on the adsorption and electron-transfer capacity at the interface of oxidized carbon nanotubes. Chem Eng J 388. 10.1016/j.cej.2020.124379

[15] Machado NCF, Jesus LAM, Pinto PS, Paula FGF, Alves MO, Mendes KHA, Mambrini RV, Barrera D, Rocha V, Santamaria R, Trigueiro JPC, Lavall RL, Ortega PFR (2021) Waste-polystyrene foams-derived magnetic carbon material for adsorption and redox supercapacitor applications. J Clean Prod 313. 10.1016/j.jclepro.2021.127903

[16] Alves MO, Ortega PFR, Carvalho FS, Braga JP (2022) Modelling the adsorption on oxidized carbon nanotubes using a statistical mechanics approach. J Mol Model 28:318. 10.1007/s00894-022-05307-4 36109369

[17] Vieira Y, Fuhr ACFP, da Silva BF, Silva LFO, Ríos ALM, Ahmad N, Irshad S, Jemli S, Dotto GL (2025) In-depth interpretations of the interactions of aromatic molecules to crosslinked surfaces using the grand canonical approach and quantum mechanics calculations. J Mol Struct 1338:142293. 10.1016/j.molstruc.2025.142293

[18] Oueslati K, Naifar A, Basha B, Lima EC, Aouaini F, Ben Lamine A (2026) Advanced statistical and stereographic analysis of hydroquinone adsorption using pine biochar: thermodynamic, energetic, and topographic perspectives. J Solution Chem 55(3):525–546. 10.1007/s10953-025-01554-0

[19] Radke CJ, Prausnitz JM (1972) Statistical mechanics of adsorption from dilute liquid solution. J Chem Phys 57(2):714–720. 10.1063/1.1678304

[20] Hill TL (1986) An introduction to statistical thermodynamics. Dover Publications, New York

[21] Lemes N, Borges E, Braga JP (2007) A general algorithm to solve linear and nonlinear inverse problems. J Braz Chem Soc 18(7):1342–1347. 10.1590/S0103-50532007000700008

[22] Hadamard J (2014) Lectures on Cauchy’s problem in linear partial differential equations. Courier Corporation, Mineola, NY

[23] Strang G (2000) Linear algebra and its applications

[24] Tikhonov AN, Goncharsky AV, Stepanov VV, Yagola AG (1995) Numerical methods for the approximate solution of ill-posed problems. Springer Dordrecht. 10.1007/978-94-015-8480-7

[25] Hopfield JJ (1982) Neural networks and physical systems with emergent collective computational abilities. Proc Natl Acad Sci 79(8):2554–2558. 10.1073/pnas.79.8.2554 PMC346238 6953413

[26] Carvalho FS, Braga JP, Alves MO (2022) Adsorbate-adsorbent potential energy function from second virial coefficient data: a non-linear Hopfield neural network approach. J Mol Model 28:286. 10.1007/s00894-022-05274-w 36056267

[27] Radke CJ, Prausnitz JM (1972) Adsorption of organic solutes from dilute aqueous solution of activated carbon. Ind Eng Chem Fundam 11(4):445–451. 10.1021/i160044a003

[28] Sun L, Zheng B, Bu C, Wei Y (2016) Moore-Penrose inverse of tensors via Einstein product. Linear Multilinear Algebra 64(4):686–698. 10.1080/03081087.2015.1083933

[29] Pandey P, Kilicman A (2024) Linear to multi-linear algebra and systems using tensors. Front Appl Math Stat 9:1259836. 10.3389/fams.2023.1259836

[30] Oueslati K, Lima EC, Ayachi F, Cunha MR, Lamine AB (2020) Modeling the removal of reactive red 120 dye from aqueous effluents by activated carbon. Water Sci Technol 82(4):651–662. 10.2166/wst.2020.347 32970618

[31] Calvete T, Lima EC, Cardoso NF, Dias SLP, Pavan FA (2009) Application of carbon adsorbents prepared from the Brazilian pine-fruit-shell for the removal of Procion Red MX 3B from aqueous solution–Kinetic, equilibrium, and thermodynamic studies. Chem Eng J 155(3):627–636. 10.1016/j.cej.2009.08.019 20398999

[32] Sghaier W, Torkia YB, Aouaini F, Alrebdi T, Lamine AB (2026) Enhancing CO adsorption and PSA refrigeration efficiency with activated carbon-graphene nanoplatelet composites: insights from statistical physics. Int Commun Heat Mass Transf 172:110644. 10.1016/j.icheatmasstransfer.2026.110644

[33] Rohatgi A (2024) WebPlotDigitizer. https://automeris.io/WebPlotDigitizer

[34] Steele WA (1973) The physical interaction of gases with crystalline solids. I. Gas-solid energies and properties of isolated adsorbed atoms. Surf Sci 36:317–352. 10.1016/0039-6028(73)90264-1

[35] Nguyen HGT, Horn JC, Thommes M, van Zee RD, Espinal L (2017) Experimental aspects of buoyancy correction in measuring reliable high-pressure excess adsorption isotherms using the gravimetric method. Meas Sci Technol 28(12):125802. 10.1088/1361-6501/aa8f83 PMC5828040 29503511

[36] Mohamed B, Qingyu Z, Moggridge GD, Abdelmottaleb BL (2018) New insight in adsorption of pyridine on the two modified adsorbents types mn200 and mn500 by means of grand canonical ensemble. J Mol Liq 263:413–421. 10.1016/j.molliq.2018.05.008

[37] Khalfaoui M, Baouab M, Gauthier R, Lamine AB (2002) Dye adsorption by modified cotton: steric and energetic interpretations of model parameter behaviours. Adsorpt Sci Technol 20(1):33–47. 10.1260/026361702760120917

[38] Mobarak M, Mohamed EA, Selim AQ, Mohamed FM, Sellaoui L, Bonilla-Petriciolet A, Seliem MK (2019) Statistical physics modeling and interpretation of methyl orange adsorption on high-order mesoporous composite of MCM-48 silica with treated rice husk. J Mol Liq 285:678–687. 10.1016/j.molliq.2019.04.116

[39] Bouaziz N, Ben Manaa M, Bouzid M, Ben Lamine A (2019) Adsorption of hydrogen in defective carbon nanotube: modelling and consequent investigations using statistical physics formalism. Mol Phys 118(3):1606460. 10.1080/00268976.2019.1606460

[40] Zhang LP, Li ZC, Wei YM, Huang HT (2023) New regularization techniques for ill-conditioning problems and their applications: choices of regularization parameters. Eng Anal Bound Elem 152:347–361. 10.1016/j.enganabound.2023.04.013

